# Different mechanisms in periventricular and deep white matter hyperintensities in old subjects

**DOI:** 10.3389/fnagi.2022.940538

**Published:** 2022-08-10

**Authors:** Jinsong Cai, Jianzhong Sun, Haiyan Chen, Ying Chen, Ying Zhou, Min Lou, Risheng Yu

**Affiliations:** ^1^Department of Radiology, Second Affiliated Hospital, Zhejiang University School of Medicine, Hangzhou, China; ^2^Department of Neurology, Second Affiliated Hospital, Zhejiang University School of Medicine, Hangzhou, China

**Keywords:** white matter hyperintensities, cerebral blood flow, vein, glymphatic pathway, mechanism

## Abstract

**Objective:**

Although multiple pieces of evidence have suggested that there are different mechanisms in periventricular white matter hyperintensities (PWMHs) and deep white matter hyperintensities (DWMHs), the exact mechanism remains uncertain.

**Methods:**

We reviewed clinical and imaging data of old participants from a local She Ethnic group. We assessed the cerebral blood flow of white matter (WM-CBF) on arterial spin-labeling, deep medullary veins (DMVs) visual score on susceptibility-weighted imaging, and index for diffusion tensor image analysis along the perivascular space (ALPS index), indicating glymphatic function on diffusion tensor imaging. Furthermore, we investigated their relationships with volumes of PWMHs and DWMHs.

**Results:**

A total of 152 subjects were included, with an average age of 63 ± 8 years old. We found that higher age and history of hypertension were independently related to higher volumes of both PWMHs and DWMHs (all *p* < 0.05). Lower ALPS index was independently associated with higher PWMHs volumes (β = 0.305, *p* < 0.001), and this relationship was accounted for by the indirect pathway *via* DMVs score (β = 0.176, *p* = 0.017). Both lower ALPS index and WM-CBF were independent risk factors for higher DWMHs volumes (β = −0.146, *p* = 0.041; β = −0.147, *p* = 0.036).

**Conclusions:**

Our study indicated that there were different mechanisms in PWMHs and DWMHs. PWMHs were mainly attributed to the damage of veins due to the dysfunction of the glymphatic pathway, while DWMHs could be affected by both ischemia-hypoperfusion and dysfunction of the glymphatic pathway.

**Advances in knowledge:**

The relationship between glymphatic dysfunction and PWMHs might be accounted for by the indirect pathway *via* venous abnormalities, a glymphatic dysfunction, and lower CBF in white matter were independent risk factors for DWMHs.

## Introduction

White matter hyperintensities (WMHs), characterized by bilateral and symmetrical hyperintensities on fluid-attenuated inversion-recovery imaging (FLAIR), are very common neuroradiological manifestations in the elderly (de Laat et al., 2011; Wardlaw et al., 2013; Graff-Radford et al., 2019). Although numerous studies have focused on the pathogenesis of WMHs, the exact mechanism was still not well-understood and could be multifactorial (Debette and Markus, 2010; Gouw et al., 2011; Wardlaw et al., 2013). Chronic ischemia-hypoperfusion induced by small artery injury is traditionally considered to be an important mechanism of WMHs. On the contrary, some scholars put forward that WMHs could be related to venous abnormalities and were mainly supported by the collagen degeneration in periventricular veins (Moody et al., 1995).

The different distribution of WMHs may explain the heterogeneity of the mechanism. Evidence from MRI and pathological comparison showed that in deep WMHs (DWMHs), the main pathological changes were demyelination, axonal cleavage, and gliosis. While in periventricular WMHs (PWMHs), the changes were the destruction of endothelium and interstitial edema (Haller et al., 2013; Shim et al., 2015). Ischemia could directly damage myelin because of its ischemic intolerance, while venous occlusion might lead to the accumulation of interstitial fluid around the ventricle by preventing its reabsorption and showing interstitial edema (Black et al., 2009; Hamilton et al., 2016). These suggest that there may be different vasculogenic mechanisms in DWMHs and PWMHs.

In addition, a recent study has reported that dysfunction of a newly discovered brain clearance system, the glymphatic pathway, played an important role in the development of both PWMHs and DWMHs (Zhang W. et al., 2021). The glymphatic pathway mainly includes the cerebrospinal fluid (CSF) influx into the brain parenchyma through the periarterial spaces, exchange with interstitial fluid (ISF) through aquaporin-4 channels on the end feet of astrocytes, and ISF outflow through the perivenous spaces (Iliff et al., 2012). However, although the glymphatic CSF clearance passes through the perivascular spaces, the exact interaction between the structure or function of the glymphatic pathway and vessels, as well as their effects on the development of PWMHs and DWMHs, is still unclear.

Therefore, in this study, we measured volumes of DWMHs and PWMHs on FLAIR, evaluated cerebral blood flow (CBF) on arterial spin-labeling (ASL) to reflect ischemia severity, assessed deep medullary veins (DMVs) visual score on susceptibility-weighted imaging (SWI) to reflect venous condition (Zhang et al., 2017), calculated the index for diffusion tensor image analysis along the perivascular space (ALPS index) on diffusion tensor imaging (DTI) to reflect glymphatic function (Taoka et al., 2017; Yokota et al., 2019; Zhang W. et al., 2021) and investigated the interaction among these pathophysiological changes and their effects on DWMHs and PWMHs volumes.

## Materials and methods

### Patients

Participants from a local She Ethnic group were recruited between May 2018 and December 2018. The detailed inclusion criteria were as follows: (1) age >40; (2) without a history of infarct (except lacunar infarction) or hemorrhage; (3) without infarct lesion restricted diffusion on current diffusion weight imaging; (4) without intracranial hemorrhage lesion on current SWI; (5) without a history of multiple sclerosis, Alzheimer's disease, Parkinson's disease, and head trauma; (6) without white matter lesions of non-vascular origin (e.g., immunological-demyelinating, metabolic, toxic, and infectious). All participants underwent multimode MRI. Demographic information and vascular risk factors including age, gender, and history of hypertension, diabetes, hyperlipidemia, and smoking were recorded. Mini-mental State Examination was used to assess cognitive function by neurologists blinded to MRI images.

### MRI protocol

All the brain examinations were performed by a 3.0T MR (GE Healthcare, USA) using an 8-channel brain phased array coil. ASL was acquired using a spin-echo pulse sequence with repetition time = 4,611 ms, echo time = 10.5 ms, inversion time = 1,525 ms, flip angle = 111°, slice thickness = 4 mm, matrix = 128 × 128, field of view = 24 cm, and voxel size = 3.49 mm^3^. ESWAN was acquired using a three-dimension multi-echo gradient-echo sequence with 8 equally spaced echoes: echo time = 4.5 ms [first echo], inter-echo spacing = 3.6 ms, repetition time = 34 ms, flip angle = 20°, slice thickness = 2 mm without slice gap, matrix = 416 × 384, and FOV = 24 cm. DTI was a single shot, diffusion-weighted spin echo-planar imaging sequence: maximum b-value = 1,000 s/mm^2^, 30 non-collinear directions, 1 vol was acquired without diffusion weighting (*b* = 0 s/mm^2^), repetition time = 4,600 ms, echo time = 69.3 ms, slice thickness = 2 mm, slice gap = 1 mm, matrix = 160 × 160, field of view = 26 cm, and voxel size = 1.625 × 1.625 × 2 mm^3^. Conventional MRI sequences included 3D-T1 (repetition time = 7.3 ms, echo time = 3 ms, flip angle = 15°, slice thickness = 1 mm without slice gap, matrix = 256 × 256, field of view = 24 cm), T2-FLAIR (repetition time = 8,000 ms, echo time = 150 ms, inversion time = 2,100 ms, flip angle =90°, slice thickness = 4 mm without slice gap, matrix = 256 × 256, field of view = 24 cm), and DWI (repetition time = 5,000 ms, echo time = 86 ms, and b value = 1,000 s/mm^2^, along three orthogonal directions, flip angle = 90°, slice thickness = 4 mm, matrix = 256 × 256, and field of view = 24 cm).

### Volume assessments of WMHs

**Figure 1:**
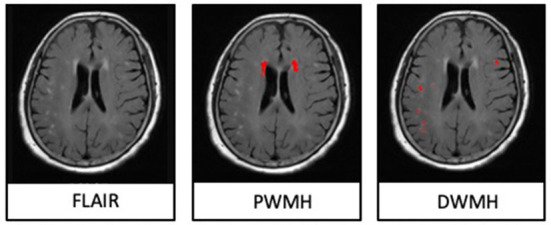
A representative case with shown final periventricular white matter hyperintensities (PWMHs) and deep white matter hyperintensities (DWMHs) segmentations on FLAIR.

The T2-FLAIR images were segmented automatically through the lesion segmentation tool (LST) in MATLAB (R2014a) pipeline integrating SPM12 (Wellcome Department of Neurology, University College of London, UK). Then, the automatically segmented lesions were manually checked and corrected on MRIcron (http://www.nitrc.org/projects/mricron) after being co-registered to the T1 images on SPM12. The manual correction was implemented as follows: (1) correction of non-white matter area being labeled as WMHs; (2) WMHs area, which was not adequately labeled as WMHs, or normal-appearing white matter falsely labeled as WMHs, and (3) division of PWMHs (10 mm away from everywhere around the lateral ventricles for confluent type) and DWMHs (Fazekas et al., 1993; Young et al., 2008). An experienced radiologist (S.J.) and an experienced neurologist (Z.Y.), who were blinded to clinical and other image data, did the manual correction together. The lesion segments were the result of their negotiation. Since the intracranial volume (ICV) of everyone was different, we corrected the WMHs/PWMHs/DWMHs volumes for further analysis as follows: (WMHs/PWMHs/DWMHs volumes × mean ICV) / ICV. The mean ICV is the mean value of ICV of all patients. Figure 1 was a representative case with shown final WMH segmentation.

### CBF measurement of white matter and gray matter

The CBF mappings were generated from ASL images on a workstation (ADW4.4, GE), then were co-registered to T1 images on SPM12. The masks of white matter and gray matter were also automatically obtained on SPM12, then manually corrected. The CBF of white matter and gray matter were then obtained from MRIcron and recorded as WM-CBF and GM-CBF.

### Assessments of DMVs visual score

**Figure 2:**
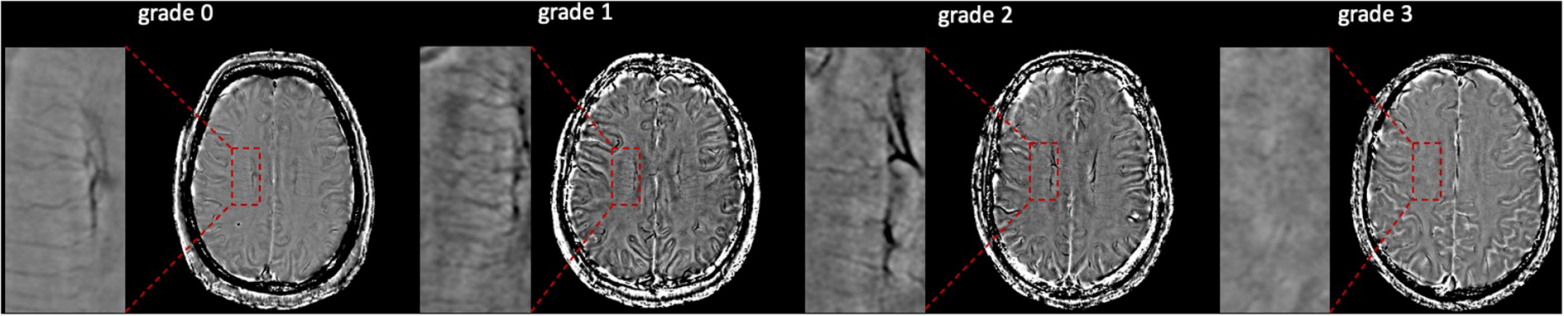
Deep medullary veins (DMVs) visual scores. An example of four-point DMV scores in parietal region: Grade 0—each vein was continuous and had homogeneous signal; Grade 1—each vein was continuous, but one or more than one vein had inhomogeneous signal; Grade 2—one or more than one vein were not continuous, presented with spot-like hypointensity; Grade 3—No observed vein was found continuous.

The assessments of DMV's visual scores were according to previous studies (Zhang et al., 2017, 2019; Zhou et al., 2020; Zhang K. et al., 2021). The ESWAN raw data were reconstructed to the magnitude and phase images on a custom-built program on a separate workstation (ADW4.4, GE). The DMVs were assessed on five consecutive periventricular slices (10-mm thick) of phase images from the level of the ventricles immediately above the basal ganglia to the level of the ventricles that immediately disappeared for each patient, considering that these slices cover most of the DMVs. According to medullary venous anatomy, six regions, including frontal, parietal, and occipital regions (bilateral, respectively), were separated into the abovementioned five slices, and the characteristics of the DMVs were then evaluated in each region, respectively. The following four-point score was used for the evaluation of DMVs in each region: Grade 0 as each vein was continuous and had a homogeneous signal; Grade 1 as each vein was continuous, but one or more than one vein had an inhomogeneous signal; Grade 2 as one or more than one vein was not continuous, presented with spot-like hypointensity; Grade 3 as No observed vein was found continuous. The final DMVs score was the sum of the six regions ranging from 0 to 18. The higher score reflects a more severe disruption of DMVs. Two neuro-radiologists (C.H. and C.Y.), who were blinded to clinical and other image data, visually assessed the DMVs scores. Figure 2 shows the representative images of DMVs' visual scores.

### Calculation of ALPS index

**Figure 3:**
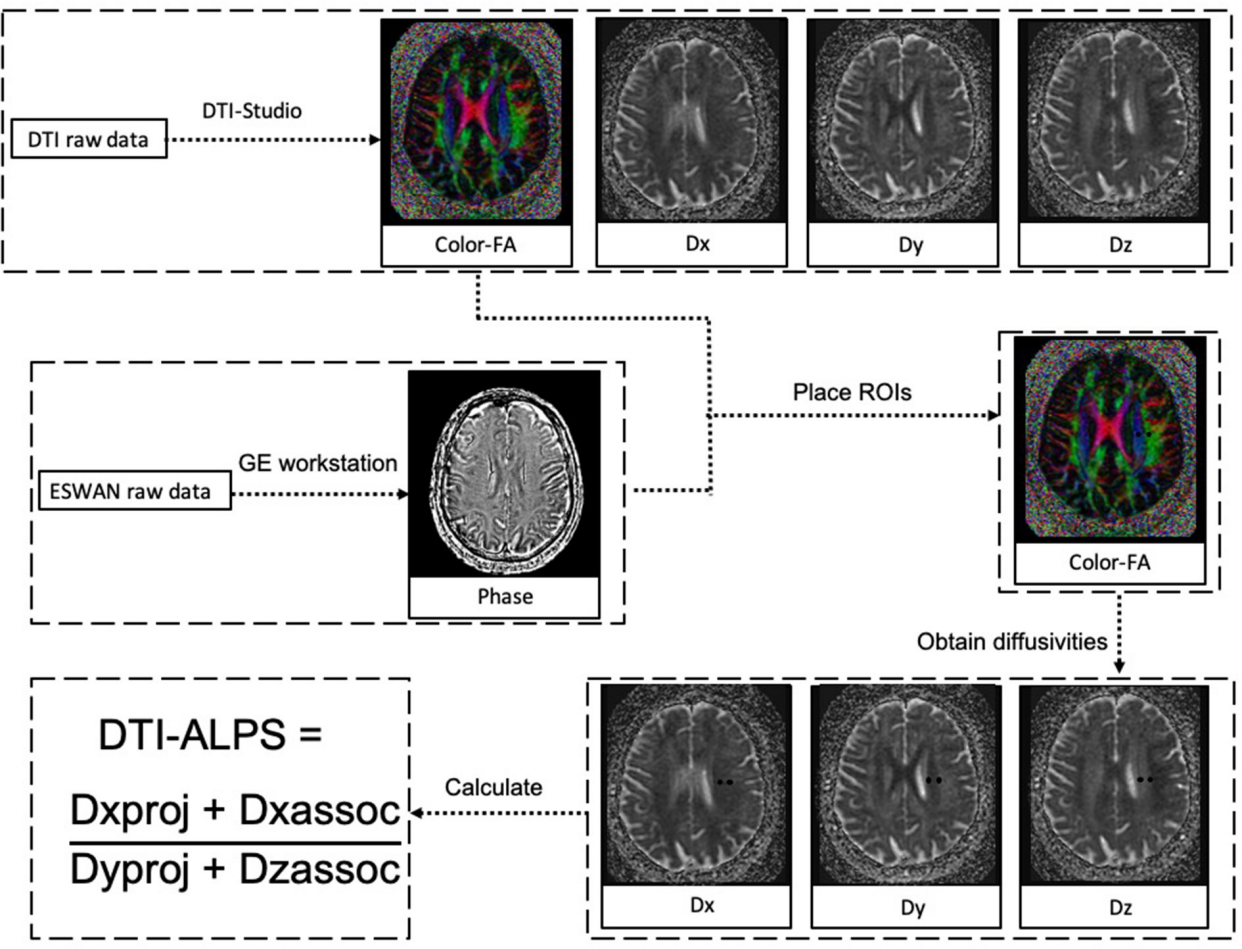
The schematic drawing of the diffusivity measurement using the index for diffusion tensor image analysis along the perivascular space (ALPS index) method. Diffusion tensor image (DTI) raw data were processed to diffusivity maps in the direction of the x-axis (Dx), y-axis (Dy), z-axis (Dz), and color-coded fractional anisotropy (FA) maps on DTI Studio. ESWAN raw data were reconstructed to phase images on a custom-built program on GE workstation. At the location where the direction of the deep medullary veins (DMVs) was vertical to ventricle body, two 5-mm-diameter regions of interest (ROIs) were placed in the area of the projection fibers and the association fibers on the color-coded FA map. The diffusivity in the directions of Dx, Dy, and Dz of ROIs on projection fibers and association fibers were recorded as Dxproj, Dyproj, Dzproj, Dxassoc, Dyassoc, and Dzassoc, respectively. Then ALPS index was calculated as [(Dxproj + Dxassoc)/(Dyproj + Dzassoc)].

Evaluation of the ALPS index was accordant with previous studies (Taoka et al., 2017; Yokota et al., 2019; Zhang W. et al., 2021). Diffusivity maps in the direction of the x-axis (Dx), y-axis (Dy), z-axis (Dz), and color-coded fractional anisotropy (FA) maps were processed on DTI Studio (https://www.mristudio.org). Two 5-mm-diameter ROIs were placed in the area of the projection fibers and the association fibers on the color-coded FA map in the location where the direction of the DMVs was vertical to the ventricle body. The diffusivity in the directions of Dx, Dy, and Dz of ROIs on projection fibers and association fibers were recorded as Dxproj, Dyproj, Dzproj, Dxassoc, Dyassoc, and Dzassoc, respectively. Then ALPS index was calculated as [(Dxproj + Dxassoc) / (Dyproj + Dzassoc)]. Figure 3 shows the schematic drawing of the diffusivity measurement using the ALPS index method.

### Statistical analysis

Statistical analysis was performed using IBM SPSS 19.0 for Windows software. Comparisons between groups were calculated by Student's *t*-test since the normalcy of the data was verified. Correlation between groups was calculated by Pearson or Spearman's test. Multivariate analysis was calculated by linear regression, and factors with a *p-value* of < 0.1 were put into the multivariate analysis models. The interobserver reliabilities of DMVs were determined using the intraclass correlation coefficient. A *p*-value of <0.05 was considered to be statistically significant.

## Results

A total of 152 subjects were included. The average age of the subjects was 63 ± 8 years old, with 77 (51%) female subjects. The average years of education were 5 ± 4.4. 103 (67.8%), 31 (20.4%), 49 (32.2%), and 37 (24.3%) subjects had a history of hypertension, diabetes mellitus, hyperlipidemia, and smoking, respectively. The average volumes of PWMHs and DAWNs were 6.4 ± 7.6 ml and 5.4 ± 6 ml, respectively. The median score of DMVs was 7 (6, 12), and the interobserver consistency of DMVs scores was 0.942. The average of WM-CBF and GM-CBF were 37.4 ± 8.4 ml/100 g-tissue/min and 41.1 ± 9.6 ml/100 g-tissue/min. The average ALPS index was 1.43 ± 0.21.

**Figure 4:**
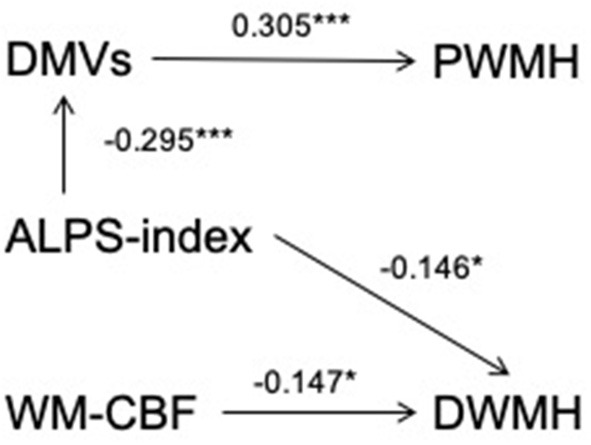
The relationship among deep medullary veins (DMVs) score, cerebral blood flow of white matter (WM-CBF) and index for diffusion tensor image analysis along the perivascular space (ALPS index) and their effects on periventricular white matter hyperintensities (PWMHs) and deep white matter hyperintensities (DWMHs). After adjusting for age, hypertension, and hyperlipidemia, a higher ALPS index was independently related to lower DMVs score (β = −0.295, *p* < 0.001), and both lower ALPS index and higher DMVs score were independently related to higher volumes of PWMHs (β = −0.141, *p* = 0.047; β = 0.305, *p* < 0.001). Furthermore, after adjusting for ALPS index, a higher DMVs score were still independently related to higher volumes of PWMHs (β = 0.176, *p* = 0.017), indicating that the relationship between ALPS index and PWMHs volumes could be accounted for by the indirect pathway *via* DMVs score. In addition, lower WM-CBF was independently related to higher volumes of DWMHs after adjusting for age, hypertension, and ALPS index (β = −0.147, *p* = 0.036), and lower ALPS index were independently related to higher volumes of DWMHs after adjusting for age, hypertension, and WM-CBF (β = −0.146, *p* = 0.041), indicating that both lower ALPS index and WM-CBF were independently risk factors for higher DWMHs volumes. *p < 0.05 and ***p < 0.001.

Table 1 shows the correlation between PWMHs and DWMHs volumes and demographic, clinical, and imaging data. Higher age, history of hypertension, DMVs score, lower WM-CBF, and lower ALPS index were related to both higher volumes of PWMHs and DWMHs (all *p* < 0.05). As Table 2 shows, higher DMVs scores and lower ALPS index were independently related to higher volumes of PWMHs after adjusting for age, hypertension, and hyperlipidemia (both *p* < 0.05). Moreover, a higher DMVs score was still independently related to higher volumes of PWMHs after further adjusting for the ALPS index (β = 0.176, *p* = 0.017). In addition, a higher ALPS index was independently related to a lower DMVs score after adjusting for age, hypertension, and hyperlipidemia (β = −0.295, *p* < 0.001). As Table 3 shows, both lower WM-CBF and lower ALPS index were independently related to higher volumes of DWMHs after adjusting for age and hypertension (both *p* < 0.05). Moreover, both lower WM-CBF and lower ALPS index were still independently related to higher volumes of DWMHs after further adjusting for ALPS index or WM-CBF (β = −0.147, *p* =0.036; β = −0.146, *p* = 0.041). In addition, WM-CBF was not independently related to ALPS index after adjusting for age and hypertension, and ALPS index was not independently related to WM-CBF after adjusting for age and hypertension (both *p* > 0.05). Figure 4 shows the relationship among DMVs score, WM-CBF, and ALPS index and their effects on PWMHs and DWMHs.

**Table 1:** Relationship between periventricular white matter hyperintensities (PWMHs) and deep white matter hyperintensities (DWMHs) volume and demographic, clinical, and imaging data.

	**PWMH volume**				**DWMH volume**			
	**Yes**	**No**	***P***	***r* (P)**	**Yes**	**No**	***P***	***r* (P)**
Female	6.2 ± 7.3	6.6 ± 8.0	0.765	-	5.2 ± 5.8	5.6 ± 6.4	0.660	-
Age (year)	-	-	-	0.495 (<0.001)	-	-	-	0.500 (<0.001)
Hypertension	7.8 ± 8.7	3.5 ± 3.2	0.001	-	6.1 ± 6.7	3.8 ± 4.0	0.025	-
Diabetes mellitus	6.4 ± 9.6	6.4 ± 7.0	0.964	-	4.8 ± 6.7	5.5 ± 5.9	0.535	-
Hyperlipidemia	7.9 ± 8.6	5.6 ± 7.1	0.088	-	5.7 ± 6.1	5.3 ± 6.1	0.702	-
Smoking	6.7 ± 9.7	6.3 ± 6.9	0.749	-	4.9 ± 6.6	5.6 ± 5.9	0.547	-
DMVs score	-	-	-	0.286 (<0.001)	-	-	-	0.161 (0.047)
CBF of white matter, mL/100 g-tissue/min	-	-	-	−0.163 (0.045)	-	-	-	−0.231 (0.004)
CBF of gray matter, mL/100 g-tissue/min	-	-	-	−0.073 (0.375)	-	-	-	−0.101 (0.218)
ALPS index	-	-	-	−0.264 (0.001)	-	-	-	−0.260 (0.001)

*DMVs, deep medullary veins; ALPS index, index for diffusion tensor image analysis along the perivascular space*.

**Table 2:** Multivariate analyses of periventricular white matter hyperintensities (PWMHs) volume.

	**β**	***P*-value**
**Model 1**		
Age (year)	0.332	<0.001
Hypertension	0.148	0.044
Hyperlipidemia	0.058	0.437
DMVs score	0.305	<0.001
**Model 2**		
Age (year)	0.464	<0.001
Hypertension	0.194	0.008
Hyperlipidemia	0.078	0.282
CBF of white matter, mL/100g-tissue/min	−0.080	0.260
**Model 3**		
Age (year)	0.448	<0.001
Hypertension	0.187	0.010
Hyperlipidemia	0.059	0.406
ALPS index	−0.141	0.047
**Model 4**		
Age (year)	0.436	<0.001
Hypertension	0.190	0.008
Hyperlipidemia	0.024	0.737
DMVs score	0.176	0.017
ALPS index	−0.089	0.220

*DMVs, deep medullary veins; ALPS index, index for diffusion tensor image analysis along the perivascular space*.

**Table 3:** Multivariate analyses of deep white matter hyperintensities (DWMHs) volume.

	**β**	***P*-value**
**Model 1**		
Age (year)	0.477	<0.001
Hypertension	0.134	0.059
DMVs score	0.085	0.229
**Model 2**		
Age (year)	0.467	<0.001
Hypertension	0.124	0.077
CBF of white matter, mL/100 g-tissue/min	−0.148	<0.037
**Model 3**		
Age (year)	0.463	<0.001
Hypertension	0.115	0.103
ALPS index	−0.147	0.041
**Model 4**		
Age (year)	0.443	<0.001
Hypertension	0.100	0.152
WM-CBF, mL/100 g-tissue/min	−0.147	0.036
ALPS index	−0.146	0.041

*DMVs, deep medullary veins; ALPS index, index for diffusion tensor image analysis along the perivascular space*.

Table 4 shows the association between PWMHs and DWMHs volumes and Mini-mental State Examination. Both PWMHs and DWMHs volumes were related to the total Mini-mental State Examination scores (both *p* < 0.05). However, after adjusting for age and years of education, only PWMHs volume was independently related to the total Mini-mental State Examination score (*p* = 0.006). In addition, both PWMHs and DWMHs volumes were independently related to scoring of attention and calculation (both *p* < 0.05), and PWMHs volume was independently related to scoring of memory (*p* = 0.004). Besides, we did not find relationships between scores of orientations, delayed recall, and language and PWMHs or DWMHs volumes (all *p* > 0.05).

**Table 4:** The association between periventricular white matter hyperintensities (PWMHs) and deep white matter hyperintensities (DWMHs) volume and Mini-mental state examination using spearman correlation and Linear regression model (adjusting for years of education and age).

**Mini-mental state examination**	**PWMHs-r (P)**	**PWMHs-β (P)**	**DWMHs-r (P)**	**DWMHs-β (P)**
Total	−0.303 (<0.001)	−0.187 (0.006)	−0.230 (0.005)	−0.089 (0.202)
Orientation	−0.137 (0.101)	−0.019 (0.830)	−0.130 (0.120)	0.036 (0.684)
Memory	−0.159 (0.057)	−0.266 (0.004)	−0.062 (0.458)	−0.007 (0.939)
Attention and calculation	−0.297 (<0.001)	−0.278 (0.001)	−0.243 (0.003)	−0.212 (0.010)
Delayed recall	−0.210 (0.011)	−0.095 (0.274)	−0.082 (0.329)	−0.013 (0.882)
Language	−0.187 (0.025)	−0.084 (0.191)	−0.125 (0.133)	0.011 (0.863)

## Discussion

The main finding of the current study is that there might be different mechanisms in PWMHs and DWMHs. Lower ALPS index and higher DMVs score were independent risk factors for higher PWMHs volumes, and the relationship of ALPS index and PWMHs volumes might be accounted for by the indirect pathway *via* DMVs score, while lower ALPS index and WM-CBF were independent risk factors for higher DWMHs volumes. In addition, both PWMHs and DWMHs volumes were independently related to the total Mini-mental State Examination score and a sub-item score of attention and calculation, as well as PWMHs volumes, were independently related to a sub-item score of memory.

We found that a higher DMVs score was an independent risk factor for higher PWMHs volumes, which was accordant with previous studies. Moody et al. (1995) found that in patients with WMHs, collagen degeneration in paraventricular venules, gradually increased with age, resulted in thickening of venous wall, stenosis of the lumen, and eventually venous occlusion. Normally, paraventricular interstitial fluid is absorbed into the perivascular space, and then flows back into the ventricle through the venous system to enter the blood circulation. However, the paraventricular interstitial fluid cannot be reabsorbed with venous occlusion and accumulates around the ventricle to form interstitial edema, which shows a high signal on FLAIR images (Black et al., 2009). Black et al. (2009) also suggested that PWMHs might be related to venous obstruction caused by venous pathological changes. Recently, Chung et al. (2014) reported that in Alzheimer's disease, jugular venous reflux might play a role in the dynamics of WMHs formation, particularly in the PWMHs, which also supported our findings. Nevertheless, it is important to note that the higher DMVs score might not simply be a risk factor but an element of a wider picture of ongoing damage of WMHs. A higher level of tissue damage can decrease the level of tissue oxygen consumption, thus, decreasing deoxyhemoglobin level in draining veins. Lower deoxyhemoglobin, in turn, could further decrease the visibility of the veins on phase images (Reichenbach et al., 1998; Essig et al., 1999; Hingwala et al., 2010).

In addition, we found that ALPS index was an independent protective factor for lower PWMHs volumes, which was also accordant with previous studies (Zhang W. et al., 2021). Interestingly, we found that the relationship of ALPS index and PWMHs volumes could be accounted for by the indirect pathway *via* DMVs score. The neuro-inflammation and Aβ deposition due to the glymphatic clearance dysfunction might be the intermediate mechanisms. A previous study has reported that the impairment of glymphatic function would decrease the clearance of inflammatory cells and cytokines and further damage the vascular endothelial cells (Steyers, 2014). Sienel et al. (2022) reported that the adhesion of leukocytes to the venous endothelium contributed to tissue damage after cerebral ischemia. Inflammation-mediated adhesion of leukocytes occurred almost exclusively in pial venules and was essentially absent in intraparenchymal vessels (Enzmann et al., 2018). Besides, glymphatic clearance dysfunction will lead to Aβ deposition, which could promote inflammatory response, as well as lead to stenosis and occlusion of cerebral microvasculature (Iliff et al., 2012; Goulay et al., 2020). Therefore, we speculated that the impairment of glymphatic function contributed to the increase of neuro-inflammation and Aβ deposition, as well as damaging and plugging postcapillary venules, which further led to the injury of the white matter of corresponding venous drainage area.

We found that both lower ALPS index and WM-CBF were independent risk factors for higher DWMH volumes. The mechanism of DWMHs caused by impairment of glymphatic function might be similar to PWMHs. That is, it mainly includes neuro-inflammation and Aβ deposition. The relationship between lower CBF and increased DWMHs might be easily explained by the susceptibility of deep white matter to ischemic damage. The deep white matter region is supplied by deep perforating arteries branching from large vessels at the base of the brain. These long perforating arteries have few branches and almost no collateral circulation. Therefore, the deep white matter is susceptible to ischemic damage (Pantoni and Garcia, 1997). An autopsy pathological study found that the density of small arteries and capillaries significantly decreased in the DWMHs area and the white matter area around the DWMHs compared with normal people, suggesting that the changes in small arteries and capillaries appeared before the white matter injury (Moody et al., 2004).

In addition, we found different effects of PWMHs and DWMHs in the decrease of cognitive functions. We found that after adjusting for age and years of education, only PWMHs were related to the total Mini-mental State Examination score, which was accordant with previous studies (Debette and Markus, 2010; Bolandzadeh et al., 2012). Cognitive function depends on intact connections within subcortical areas and between cortical and subcortical structures and may be impaired if any disruption in these connections appears. Therefore, any disruption in long white matter tracts traversing from periventricular areas may initially reduce the axonal transmission speed, and, later, cause total cognitive function impaired (van den Heuvel et al., 2006). The relationships of the cognitive domains (i.e., orientation, memory, attention and calculation, delayed recall, and language) and PWMHs/DWMHs in our data are not all consistent with previous studies. It may be due to the difference in methods (e.g., MRI sequences, the assessment methods of PWMHs and DWMHs, the neuropsychological tests, and the modifying effect of cardiovascular risk factors) and the population heterogeneities. Further studies are still needed.

The main strength of the study is to analyze DWMHs and PWMHs in a single MRI-based study. Numerous previous studies focused on the mechanisms of differences in DWMHs and PWMHs but rarely investigated them in one cohort. Limitations also needed to be considered. First, our study is retrospective with a moderate number of subjects. Second, the relatively low signal-to-noise ratio of SWI images could affect the assessment of DMVs. Moreover, the contrast of DMVs could be affected by the partial volume effect of SWI images due to the small size of DMVs, which could also affect the assessment of DMVs. Third, the low resolution of diffusion data and ASL could affect the accuracy of results. For instance, the measurements of periventricular CBF, with 4-mm slice thickness of ASL, are prone to greater errors due to partial volume effects from neighboring ventricles and gray matter structures (e.g., caudate nucleus). Besides, although we placed ROIs on one slice, the reliability of the ALPS index might still be affected since the diffusion-weighted EPI acquisition had slice gaps of 1 mm. Therefore, further study with higher resolution is needed to confirm the conclusions. Fourth, lacking pathology and follow-up images, the finding of the current study still needs further investigation. Fifth, we did not assess the internal carotid or large intracranial artery in current subjects, which might influence the result and should be considered, although we excluded subjects with a history of moderate to severe stenosis or occlusion of an internal carotid or large intracranial artery. Lastly, the Mini-mental State Examination score is relatively high for a current population with relatively low education. Post-school education might explain this. Future Study with different education level is needed to further confirm our findings.

In conclusion, there might be different mechanisms in PWMHs and DWMHs. PWMHs were mainly attributed to damage to veins due to the dysfunction of the glymphatic pathway, while DWMHs could be affected by both ischemia-hypoperfusion and dysfunction of the glymphatic pathway.

## Data availability statement

The original contributions presented in the study are included in the article/supplementary material, further inquiries can be directed to the corresponding authors.

## Ethics statement

The studies involving human participants were reviewed and approved by human Ethics Committee of the Second Affiliated Hospital of Zhejiang University. The patients/participants provided their written informed consent to participate in this study.

## Author contributions

JC, JS, and HC: conceptualization, methodology, data collection, image analysis, and writing—original draft preparation. YC and YZ: data collection and image analysis. ML: data collection. RY: conceptualization, methodology, data collection, reviewing, and editing. All authors contributed to the article and approved the submitted version.

## Conflict of interest

The authors declare that the research was conducted in the absence of any commercial or financial relationships that could be construed as a potential conflict of interest.

## Publisher's note

All claims expressed in this article are solely those of the authors and do not necessarily represent those of their affiliated organizations, or those of the publisher, the editors and the reviewers. Any product that may be evaluated in this article, or claim that may be made by its manufacturer, is not guaranteed or endorsed by the publisher.
